# Building the foundation for universal healthcare: Academic family medicine’s ability to train family medicine practitioners to meet the needs of their community across the globe

**DOI:** 10.4102/phcfm.v14i1.3506

**Published:** 2022-08-16

**Authors:** Esther M. Johnston, Nath Samaratunga, Ramakrishna Prasad, Bassim Birkland, Klaus B. von Pressentin, Shailendra Prasad

**Affiliations:** 1 National Family Medicine Residency Program, HealthPoint, The Wright Center, Auburn, United States of America; 2 Rutgers Robert Wood Johnson Medical School, Piscataway, United States of America; 3 PCMH Restore Health, Bangalore, India; 4 Academy of Family Physicians of India, Bengaluru, India; 5 Department of Community and Family Medicine, School of Public Health, University of Zambia, Lusaka, Zambia; 6 Division of Family Medicine, School of Public Health and Family Medicine, Faculty of Health Sciences, University of Cape Town, Cape Town, South Africa; 7 Department of Family Medicine and Community Health, Medical School, University of Minnesota, Minneapolis, United States of America; 8 Center for Global Health and Social Responsibility, University of Minnesota, Minneapolis, United States of America

## Abstract

**Background:**

The Declaration of Astana marked a revived global interest in investing in primary care as a means to achieve universal healthcare. Family medicine clinicians are uniquely trained to provide high-quality, comprehensive primary care throughout the lifespan. Yet little focus has been placed on understanding the needs of family medicine training programs.

**Aim:**

This study aims to assess broad patterns of strengths and resource challenges faced by academic programs that train family medicine clinicians.

**Methods:**

An anonymous online survey was sent to family medicine faculty using World Organization of Family Doctors (WONCA) listservs.

**Results:**

Twenty-nine representatives of academic family medicine programs from around the globe answered the survey. Respondents cited funding for the program and/or individual trainees as one of either their greatest resources or greatest limitations. Frequently available resources included quality and quantity of faculty and reliable clinical training sites. Frequently noted limitations included recruitment capacity and social capital. Over half of respondents reported their program had at some point faced a disruption or gap in its ability to recruit or train, most often because of loss of government recognition. Reflecting on these patterns, respondents expressed strong interest in partnerships focusing on faculty development and research collaboration.

**Lessons learnt:**

This study provides a better understanding of the challenges family medicine training programs face and how to contribute to their sustainability and growth, particularly in terms of areas for investment, opportunities for government policy and action and areas of collaboration.

**Keywords:**

family medicine; primary care; medical education; global health; community medicine.

## Introduction

The 40th anniversary of the Alma Ata declaration in 2018 saw a renewed dedication to primary health care as the ‘cornerstone of a sustainable health system for universal health coverage (UHC)’.^1,2^ The World Health Organization has emphasised that strong primary health care systems must be comprehensive and holistic, caring for people throughout their lifespan in the context of their communities and broader environment.^3^ Family medicine is a clinical primary care discipline in which healthcare providers are trained to realise the ideals of comprehensive primary care.^4,5^ However, investment and support of family medicine training programs from both institutions and governments have been subpar.^6,7^

Previous surveys have compared the curricular design and status of family medicine training programs regionally and globally.^8,9,10,11^ Studies have also assessed resources and barriers to faculty development, specifically in the African region.^12,13^ However, limited attention has been given to understanding the strengths and challenges faced by academic centres that train family physicians globally. This study aims to address this.

## Methods

The authors, members of the Family Medicine Global Education Network (FamMedGEN), designed a survey to describe the program structure, training capacity and limitations of academic departments of family medicine around the world, as well as possible opportunities for partnership and collaboration between departments. The survey instrument was created through discussion and literature review by the research team. An initial draft of the survey was pilot tested with five key informants from four countries. Subsequently, the revised survey tool (Appendix 1) was uploaded to the Survey Monkey platform and sent via e-mail and WhatsApp through the World Organization of Family Doctors (WONCA) Working Parties on Education and Research and WONCA Africa listservs. These listservs are composed of family medicine physicians around the world who express interest in the subject content; they do not require membership to join. The survey was open from 01 June 2021 to 17 August 2021. Respondents remained anonymous, with the only identifying information requested by the program being geographic region.

This research was approved under the Human Research Protections Program (HRPP) of the University of Minnesota Institutional Review Board.

### Ethical considerations

The Human Research Protections Program (HRPP) of the University of Minnesota Institutional Review Board reviewed this proposed study and determined that the proposed activity is not research involving human subjects as defined by DHHS and FDA regulations (ref. no. STUDY00011867).

## Results

### Training program structure

Twenty-nine individuals responded to the survey, with all geographic regions represented (Appendix 2). The results were analysed by response frequency. Most respondents represented university-based programs, either in large academic health centres (48.3%) or in the community (37.9%). Remaining respondents represented programs at community health centres or regional hospitals. Most respondents (72.4%) were associated with programs in existence for greater than 10 years.

In describing faculty composition, some responses were either left blank or did not sum to 100%; these responses were removed from the survey results (Appendix 2). In a majority (65.5%) of the programs, more than 50% of faculty members were family medicine specialists or general practitioners, as opposed to other speciality clinicians or physicians, with 14 (48.3%) of respondents reporting that above 90% of their training program faculty were family medicine specialists.

The number of respondents reporting that their programs offer family medicine training at the undergraduate level was equivalent to those offering graduate-level training (86.2%).

### Training program capacity

Twenty-two respondents (75.9%) provided complete responses to questions related to available resources at their training program (Table 1). Resources frequently cited as top strengths of programs included quality (59.0%) and quantity (31.8%) of faculty, funding for the program as a whole (31.8%) and for individual students or trainees (50.0%), and reliable clinical sites (31.8%).

**TABLE 1:** Survey results – Program resources and limitations identified by survey respondents.

Resources to support training	% of respondents ranking in top 3 for largest supply		% of respondents ranking in top 3 for greatest limitation	
	*n* = 22	%	*n* = 22	%
High-quality faculty	13	59.0	4	18.2
Student or trainee funding	11	50	10	45.5
Program funding	7	31.8	13	59.1
Reliable clinical training sites	7	31.8	4	18.2
Quantity of faculty	7	31.8	5	22.7
Availability of senior faculty for mentorship and modeling	5	22.7	5	22.7
Social capital (e.g. strong relationships with clinical sites, government relationships, relationships with other family medicine departments)	4	18.2	8	36.4
Access to textbooks and online resources	3	13.6	0	0
Recruitment capacity	3	13.6	9	40.9
Internal research capacity	3	13.6	4	18.2
Faculty continuing medical education and continuing professional development	2	9.0	1	4.5
Access to scientific journals	1	4.5	1	4.5

Note: Other: *n* = 2 (9.0%) – ‘time to do research’, ‘COVID has impacted face to face’.

While some respondents saw these resources as adequate, others noted program funding and individual student or trainee funding among the top resource limitations (at 59.1% and 45.5%, respectively). Other top limitations included recruitment capacity (40.9%) and social capital (36.4%). Examples of social capital included strong relationships with clinical sites, governmental bodies and/or other family medicine departments.

Fifteen respondents (51.7%) reported their program had experienced a disruption in its ability to recruit or train residents at some point during its life cycle (Appendix 3). The most frequent source of the disruption was the loss of government recognition for the program (40.0%).

### Opportunities for partnership

**FIGURE 1:**
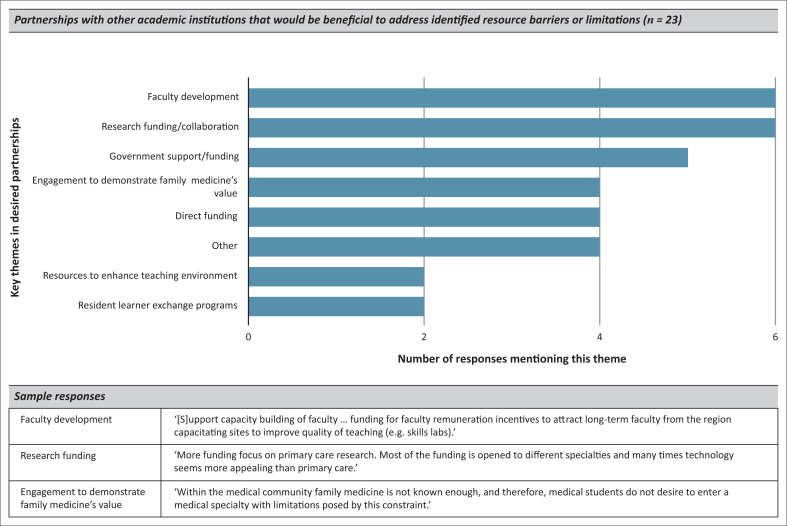
Survey results – Desired academic partnerships.

Twenty-three (79.3%) respondents provided suggestions of academic partnerships that might be beneficial to address the resource barriers or limitations they had identified (Figure 1). The most frequently suggested benefits from partnerships included a focus on faculty development and research as well as collaborations to improve government support, demonstrate family medicine’s overall value and provide direct funding. Partnerships focusing on learner exchange and/or providing enhancements in the teaching environment (examples provided included simulation training and access to journals and textbooks) were cited less commonly.

## Discussion

### Impact

It is important to consider the capacity of academic family medicine to adequately produce the primary care workforce needed to ensure universal healthcare. This survey highlights the most common strengths and resources that family medicine training programs note in abundance, including quality and quantity of faculty, program and trainee funding and reliable clinical sites. Meanwhile, recruitment capacity and social capital were common limitations and barriers. Programs were divided as to whether funding for students or trainees was their most dependable resource or their greatest challenge. These findings provide insight into the existing capacity of training programs and highlight potential areas for investment.

Over half of respondents noted their program had at some point experienced a disruption in their ability to train, with loss of government recognition identified most frequently as the main cause. This suggests that investment in primary care should be valued as a long-term policy goal, as gaps in training can potentially disrupt an academic unit’s long-term viability.

Respondents most frequently suggested that partnerships with other academic institutions would be most beneficial if they were focused on faculty development and research, while learner exchange and direct resource provision were considered less beneficial. As institutions reconsider how to design more equitable, multidirectional global partnerships, these responses highlight key areas in which to consider collaboration.

This study was designed to provide a high-level overview of challenges that academic family medicine departments face. For family medicine to thrive, there is a need for further studies that examine governmental support of academic family medicine departments in various countries, the specific variations of academic department functioning and areas in which academic departments could benefit from robust partnerships with professional organisations like WONCA, among other topics.

### Limitations

This study offers a cross-sectional view into the current state of family medicine training globally, with respondents representing a broad distribution of geographic regions. This was achieved through utilising established WONCA listservs for family medicine educators. However, as the survey was forwarded to a regional WONCA-specific listserv for Africa, this may have resulted in over-sampling of that region. An added bias may have been introduced by the survey’s language, as it was offered only in English. Additionally, as more established training programs may be better connected to WONCA and its working groups, the survey may have over-sampled more long-standing training programs (21 of the 29 respondents represented training programs in existence for > 10 years).

The survey intentionally did not ask respondents to provide the name or even the country of their program, to ensure anonymity (recognising that some countries have only one family medicine training program) and facilitate transparency in response. However, it is possible that anonymising respondents in this way may have led to over-sampling of some programs.

Finally, this survey addresses programs’ self-perceived resources and barriers to develop family medicine providers capable of meeting the unique needs of their communities. It is important to note that learners in these programs – and perhaps the community itself – might assess that balance differently.

## Conclusion

As policymakers and potential global partners continue to pursue a path towards cementing support for primary health care as the basis for universal healthcare, it is critical to consider how best to support family medicine training programs that produce highly skilled clinical leaders at the community level. This survey provides an opportunity to gain a better understanding of what challenges are faced and how best to contribute to the sustainability and growth of these programs.

## Appendix 1: Survey tool

Primary care is an important and essential part of the health workforce of a country. Family medicine (or general practice) is the main medical discipline that represents primary care. As we look at the future of healthcare in various populations served, the global conversation of universal healthcare is urging us to create more family physicians. The purpose of this survey is to gauge the current capacity within your academic institution to adequately address the need for producing more family physicians to serve your community.

The development of family medicine is dependent on academic institutions, adequate practice settings that hire family medicine graduates and policy aspects that emphasise the need for family medicine training.

The insights gleaned from this study will be shared with governments, family medicine academies and institutions to guide the growth of the discipline. The results will also be used to facilitate partnerships between academic institutions to support each other in the growth of the discipline. For the purposes of this survey, ‘family doctor’ is defined as a physician who provides holistic primary care across a wide spectrum of patients and is rooted in the community and their needs. For the purposes of this survey, ‘faculty’ is defined as a member of an academic institution who aids in student education and training.

Disclaimer: Responses to this survey will remain anonymous and IP addresses will not be collected. This survey is being conducted by the Family Medicine Global Education Network.

### Program structure

1. Which of the following structures most accurately describes your department structure?
  [ ] University-based or large academic health centre-based
  [ ] University- or community-based
  [ ] Community health centre-based
  [ ] District or regional hospital-based
2. For how long has your training program been in existence?
  [ ] 0–2 years
  [ ] 3–7 years
  [ ] 8–10 years
  [ ] More than 10 years
3. What percentage of your faculty body are…
  Family medicine specialists or general practitioners (%): _______
  Other speciality clinicians or physicians (%): _______
4. What percentage of your faculty body are…
  Early career (0–5 years of experience) (%): _______
  Mid-career (6–10 years of experience) (%): _______
  Late career (11+ years of experience) (%): _______
5. Does your program offer… (Check all that apply)
  [ ] Training experiences in family medicine for students enrolled in undergraduate medical education
  [ ] Specialised graduate medical education in family medicine
  [ ] Formal training experiences for other cadres of health providers (nurse practitioners, physician assistants, etc.)
  [ ] None of the above

### Capacity

6 How would you rank resources for your department in terms of supply, with 1 being the highest? (What resources do you have more of?)
  ___ Program funding
  ___ Student or trainee funding
  ___ Reliable clinical training sites
  ___ High-quality faculty
  ___ Quantity of faculty
  ___ Availability of senior faculty for mentorship or modeling
  ___ Access to scientific journals
  ___ Access to textbooks and online resources
  ___ Recruitment capacity
  ___ Internal research capacity
  ___ Social capital (e.g. strong relationships with clinical sites, government relationships, relationships with other family medicine departments)
  ___ Faculty continuing medical education or continuing professional development
7 What would you say are the top three resource limitations your department faces in providing family medicine or general practitioner training? (Select 3)
  ___ Program funding
  ___ Student or trainee funding
  ___ Reliable clinical training sites
  ___ High-quality faculty
  ___ Quantity of faculty
  ___ Availability of senior faculty for mentorship or modeling
  ___ Access to scientific journals
  ___ Access to textbooks and online resources
  ___ Recruitment capacity
  ___ Internal research capacity
  ___ Social capital (e.g. strong relationships with clinical sites, government relationships, relationships with other family medicine departments)
  ___ Faculty continuing medical education or continuing professional development
  ___ Other (please specify)
8 What kinds of partnerships with other academic institutions would be beneficial to address the resource barriers or limitations you identified in Question 7?
9 Has your department or program ever faced a disruption or gap in its ability to recruit or train residents?
  [ ] Yes
  [ ] No
10 If you answered ‘yes’ to Question 9, what was the source of the disruption? If no, select ‘N/A’ *(check all that apply)*.
  [ ] Loss of national or speciality accreditation
  [ ] Loss of funding for student stipends
  [ ] Loss of funding for faculty
  [ ] Loss of training facilities
  [ ] Loss of government recognition
  [ ] N/A
  [ ] Other (please specify)
11 In what geographic region is your department or school?
  [ ] Africa
  [ ] Asia
  [ ] Central and Eastern Europe
  [ ] Oceania
  [ ] Mediterranean and Middle East
  [ ] North America
  [ ] South America
  [ ] Western Europe

## Appendix 2: Survey results – structure of programs represented by survey respondents

**Table:**

Survey results	*n*	%
**Geographic region of respondent (*n* = 29)**		
Africa	8	27.6
Asia	4	13.8
Central and Eastern Europe	2	6.9
Mediterranean and Middle East	1	3.5
North America	3	10.3
Oceania	1	3.5
South America	4	13.8
Western Europe	2	6.9
**Department structure (*n* = 29)**		
University or large academic health centre-based	14	48.3
University- or community-based	11	37.9
Community health centre-based	1	3.5
District or regional hospital-based	5	17.2
**Length of training program existence (*n* = 29)**		
0–2 years	2	6.9
3–7 years	3	10.3
8–10 years	3	10.3
> 10 years	21	72.4
**Family medicine specialists or general practitioners as composition of faculty body (*n* = 24)**		
100%	7	24.1
90% – 99%	7	24.1
50% – 89%	5	17.2
11% – 49%	1	3.5
≤ 10%	4	13.8
**% Faculty by career stage (*n* = 23)**		
	**Range**	**Average**
Early career (0–5 years experience)	0% – 99%	30.7%
Mid-career (6–10 years experience)	0% – 60%	30.0%
Late career (11+ years experience)	0% – 95%	39.4%
**Program offerings (*n* = 29)**		
Training experiences in family medicine for students enrolled in undergraduate education	25	86.2
Specialised graduate medical education in family medicine	25	86.2
Formal training experiences for other cadres of health providers (nurse practitioners, physician assistants, etc.)	8	27.6
None of the above	0	0.0

## Appendix 3: Survey results – prevalence and causes of program training disruption

**Table:**

% Respondents reporting their program had ever faced a disruption or gap in its ability to recruit or train residents (*n* = 29)		
Yes	15	51.7%
No	14	48.3%
Source cited for the reported disruption (*n* = 15) – multiple responses allowed		
Loss of national or speciality accreditation	1	6.7%
Loss of funding for student stipends	1	6.7%
Loss of funding for faculty	2	13.3%
Loss of training facilities	1	6.7%
Loss of government recognition	6	40.0%
N/A or no response	8	53.3%
